# Integrating Enhanced HIV Pre-exposure Prophylaxis Into a Sexually Transmitted Infection Clinic in Lilongwe: Protocol for a Prospective Cohort Study

**DOI:** 10.2196/37395

**Published:** 2022-12-05

**Authors:** Sarah E Rutstein, Mitch Matoga, Jane S Chen, Esther Mathiya, Beatrice Ndalama, Naomi Nyirenda, Naomi Bonongwe, Shyreen Chithambo, Maganizo Chagomerana, Gerald Tegha, Mina C Hosseinipour, Michael E Herce, Edward Jere, Robert G Krysiak, Irving F Hoffman

**Affiliations:** 1 Division of Infectious Diseases Department of Medicine University of North Carolina at Chapel Hill Chapel Hill, NC United States; 2 University of North Carolina Project Malawi University of North Carolina Lilongwe Malawi; 3 District Health Office Ministry of Health Lilongwe Malawi

**Keywords:** pre-exposure prophylaxis, PrEP, sexually transmitted infections, STI, sub-Saharan Africa, partner notification

## Abstract

**Background:**

Pre-exposure prophylaxis (PrEP) reduces HIV acquisition risk by >90% and is a critical lever to reduce HIV incidence. Identifying individuals most likely to benefit from PrEP and retaining them on PrEP throughout HIV risk is critical to realize PrEP’s prevention potential. Individuals with sexually transmitted infections (STIs) are an obvious priority PrEP population, but there are no data from sub-Saharan Africa (SSA) confirming the effectiveness of integrating PrEP into STI clinics. Assisted partner notification may further enhance STI clinic–based PrEP programming by recruiting PrEP users from the pool of named sexual partners of individuals presenting with an incident STI. However, the acceptability, feasibility, and effectiveness of these integrated and enhanced strategies are unknown.

**Objective:**

This study aims to describe the implementation outcomes of acceptability, feasibility, and effectiveness (regarding PrEP uptake and persistence) of integrating an enhanced PrEP implementation strategy into an STI clinic in Malawi.

**Methods:**

The enhanced PrEP STI study is a prospective cohort study enrolling patients who are eligible for PrEP (aged ≥15 years) who are seeking STI services at a Lilongwe-based STI clinic. Data collection relies on a combination of in-depth interviews, patient and clinic staff surveys, and clinic record review. All enrolled PrEP users will be screened for acute HIV infection and receive quarterly testing for *Neisseria gonorrhea*, *Chlamydia trachomatis*, and syphilis. Participants will be asked to name recent sexual partners for assisted notification; returning partners will be screened for PrEP eligibility and, if interested, enrolled into the cohort of PrEP initiators. We will also enroll patients who are eligible for PrEP but choose not to initiate it, from the STI clinic. Patient participants will be followed for 6 months; we will assess self-reported PrEP use, PrEP refills, sexual behaviors, perceived HIV risk, and incident STIs. Clinic staff participants will be interviewed at baseline and at approximately 6 months and will complete surveys examining the perceived acceptability and feasibility of the integrated and enhanced PrEP strategy.

**Results:**

Enrollment began in March 2022 and is projected to continue until February 2023, with patient participant follow-up through August 2023. The results of this study are expected to be reported in 2024.

**Conclusions:**

This study will generate important evidence regarding the potential integration of PrEP services into STI clinics in SSA and preliminary data regarding the effectiveness of an enhanced intervention that includes assisted partner notification as a strategy to identify potential PrEP users. Furthermore, this trial will provide some of the first insights into STI incidence among PrEP users recruited from an STI clinic in SSA—critical data to inform the use of etiologic STI testing where syndromic management is the current standard. These findings will help to design future PrEP implementation strategies in SSA.

**Trial Registration:**

ClinicalTrials.gov NCT05307991; https://clinicaltrials.gov/ct2/show/NCT05307991

**International Registered Report Identifier (IRRID):**

DERR1-10.2196/37395

## Introduction

### Background

Daily oral pre-exposure prophylaxis (PrEP) reduces HIV acquisition risk by 90% [1-6]. HIV prevention policies in sub-Saharan Africa (SSA), including Malawi, increasingly include PrEP as an evidence-based intervention to decrease HIV incidence [7-9]. Identifying individuals most likely to benefit from PrEP and helping them to navigate complex, dynamic obstacles to uptake and retention are key to maximize HIV prevention. Ultimately, PrEP persistence (ie, continued engagement in PrEP care and drug adherence) for individuals at greatest risk of HIV acquisition is needed to maximize PrEP outcomes [10].

PrEP screening frequently uses epidemiologic risk profiles and relies on self-reported risk behaviors, such as sex work or identifying as a man who has sex with men, which are frequently stigmatized and underreported, particularly in SSA [11-18]. Risk scores may perform well in identifying individuals at high risk of HIV infection, but generally require self-identification as a member of a key population, which may limit their utility where these classifications are stigmatized. In contrast, an incident sexually transmitted infection (STI) is an indicator of unprotected sex and, in high HIV prevalence settings, a reasonable proxy for risk of HIV exposure. Incorporating PrEP with STI care is an efficient means of leveraging clinic infrastructure and an appealing opportunity to integrate related services while adding value for PrEP users [19]. Illustrating the importance of including STI screening with PrEP programs, multiple studies have observed high rates (>30%) of incident STIs while on PrEP [20,21], and there is mounting evidence that STI incidence increases after starting PrEP [22-27]. Although the World Health Organization (WHO) includes individuals with STIs as a priority PrEP population [28], no previous study has combined PrEP with existing STI clinics in SSA, and the acceptability and feasibility among patients at the STI clinic and clinic staff (ie, clinicians and counselors) regarding the integration of these services remains unknown.

Furthermore, partners of PrEP users, particularly PrEP users with a recently diagnosed STI, may benefit from PrEP services. Assisted partner notification (aPN) is a WHO-endorsed strategy in which HIV-infected index cases name recent sexual partners, who are subsequently contacted and offered HIV testing services [29]. This extremely effective and efficient strategy has historically been deployed specifically for targeted HIV case finding [30] or, more recently, among men who have sex with men in Kenya as a strategy to link HIV-uninfected partners of individuals diagnosed with HIV to PrEP services [31]. Understanding the acceptability and feasibility of extending aPN as an approach to reach high-risk sexual networks of PrEP users who may not otherwise be linked to HIV prevention services could expand PrEP’s reach and effectiveness.

Connecting individuals at high risk of HIV to PrEP is only the first step to improve PrEP effectiveness. Suboptimal adherence [20,32] and premature discontinuation despite ongoing HIV risk [33-40] drastically limits PrEP’s prevention potential, with 50% to 90% of users stopping PrEP within 6 months [40-44]. Discrepancies between perceived and actual risk are well documented in Malawi, an east African country with a generalized HIV epidemic (adult HIV prevalence of approximately 9%) [45,46]. Integrating etiologic STI testing with PrEP affords providers and PrEP users an objective indicator of risk, thus facilitating tailored PrEP counseling that can address risk misperception and potentially helping to align *perceived* HIV risk with *actual* HIV risk to further optimize PrEP persistence [47,48-52]. Unfortunately, largely owing to resource constraints, Malawi, similar to most of SSA, currently does not offer STI testing, relying instead on syndromic screening for STIs, thereby missing a significant number of asymptomatic infections [53-57]. Understanding how or if incident STIs influence PrEP counseling, affect perceived risk, and possibly improve risk-aligned PrEP persistence is critical to refine PrEP management guidelines in the region.

Efficiently and effectively scaling up PrEP in Malawi requires identifying the right individuals to start PrEP (based on the risk of HIV acquisition) and developing strategies to improve PrEP persistence for as long as those individuals remain at increased risk of HIV. Local guidelines recommend that individuals seeking STI clinical services are appropriate for PrEP, presuming that they meet other eligibility criteria. The Malawi Ministry of Health (MOH) has recently started to offer PrEP at an urban STI clinic in Lilongwe, Malawi, one of the first times this integrated approach has been deployed in the region. Diversifying PrEP delivery models, including adjusting the location and associated service packages, may improve uptake of and persistence on PrEP [58-61].

### Objectives

By recruiting participants from STI clinics, we will identify a PrEP-eligible population using objective evidence of recent sexual risk (STI), regardless of self-identification as a member of a key population, and explore the use of aPN as a strategy to recruit additional PrEP users. The overarching objective of this proposal is to examine the feasibility, acceptability, and effectiveness (ie, PrEP uptake and PrEP persistence) of integrating PrEP into an STI clinic in Malawi, including an evaluation of enhanced PrEP services via recruitment of recent sexual partners for possible PrEP initiation and provision of etiologic STI testing alongside PrEP care.

## Methods

### Study Overview

**Figure 1:**
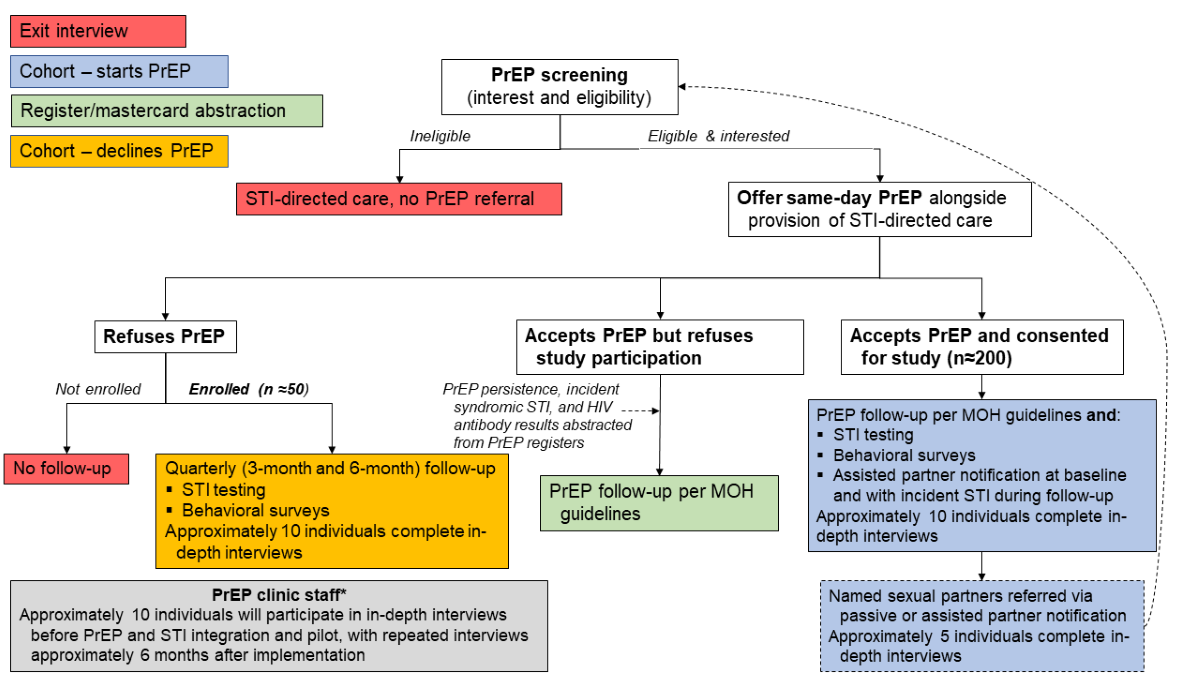
Study flow diagram for persons seeking STI services.

In this prospective pilot cohort study, we will explore the feasibility, acceptability, and effectiveness of an enhanced PrEP implementation strategy integrated into an STI clinic in Lilongwe, Malawi. Our enhanced strategy pairs STI clinic–based PrEP distribution with aPN and etiologic STI testing. We will enroll individuals initiating PrEP at the Bwaila District Hospital STI clinic. Bwaila STI is a public STI clinic in central Lilongwe, Malawi, with approximately 15,000 patient visits annually. In this study, we will follow incident STIs, self-reported risk behaviors, perceived HIV risk, and PrEP use for 6 months (Figure 1). These index participants will be asked to name and provide locator information about recent sexual partners. Partners who do not return to the clinic within 14 days will be traced and, if interested and eligible, will be offered PrEP and enrollment into the prospective cohort. We will also enroll a small subset of PrEP-eligible individuals seeking care at the STI clinic who decline PrEP, following similar biomedical and behavioral outcomes for 6 months.

We will contextualize acceptability and feasibility outcomes through quantitative and qualitative analyses; specifically validated implementation outcome measures (Acceptability of Intervention Measure and Feasibility of Intervention Measure) [62]; and in-depth interviews with STI clinic staff involved in the provision of PrEP or related services (ie, aPN and STI testing), index patients, and referred partners. All basic PrEP services, including the PrEP medications, are provided by the Malawi MOH. Currently, the only approved PrEP in Malawi is daily tenofovir disoproxil fumarate and lamivudine or tenofovir disoproxil fumarate and emtricitabine—intermittent (so-called event driven) PrEP is not endorsed by Malawi guidelines. Our protocol was designed to easily adapt if other PrEP agents are approved or offered at the enrolling clinic, including long-acting injectable (LAI) PrEP. Findings of this study will inform future implementation trials examining integrated services as an implementation strategy to improve PrEP uptake and persistence.

### Study Populations and Eligibility Criteria

Participants in this study are of two types: (1) potential PrEP users (including referred sexual partners) and (2) STI clinic staff (Textbox 1). PrEP eligibility will be defined according to current Malawi guidelines (Textbox 2). If these guidelines change during the study, our eligibility will be updated to reflect the modified guidelines. Clinic staff members, including STI nurses, PrEP nurses, HIV counselors, and clinic management, who are engaged in the provision of PrEP (including screening and referral), aPN, or STI services will be eligible for feasibility and acceptability evaluations (Textbox 1).

**Eligibility criteria for sexually transmitted infection (STI) clinic patient (potential pre-exposure prophylaxis [PrEP] user) participants and clinic staff participants.**

**Inclusion criteria for potential PrEP user (patient)**

- Aged ≥15 years
- Eligible for PrEP according to Malawi PrEP guidelines (refer to Textbox 2)
- Presenting for care at STI clinic (primary presentation or referral from partner based on STI or HIV exposure)
- Able to consent for study participation and willing to provide locator information for follow-up tracing

**Exclusion criteria for potential PrEP user (patient)**

- Current imprisonment or incarceration in a medical or psychiatric facility

**Inclusion criteria for STI clinic staff**

- Aged ≥18 years
- Involved in duties relevant to integration or provision of PrEP or assisted partner notification at STI clinic

**Exclusion criteria for STI clinic staff**

- Unable or unwilling to provide informed consent

**Malawi Ministry of Health—pre-exposure prophylaxis (PrEP) eligibility criteria.**

- Aged ≥15 years
- HIV seronegative
- At substantial risk for HIV, with prioritization of the following individuals:
  People who buy or sell sex
  Key population (female sex workers, men who have sex with other men, and transgender individuals)
  Vulnerable population including adolescent girls and young women aged 15-24 years
  Clients with sexually transmitted infection
  Serodiscordant couples including HIV-negative women who are pregnant or breast feeding or HIV-negative men or women for whom their HIV-infected partner is not on antiretroviral therapy (ART), is on ART for <6 months, has an unsuppressed or high viral load, or is nonadherent to ART
- Have ruled out acute HIV infection or defer PrEP initiation for anyone with signs or symptoms consistent with acute HIV infection
- Willingness to attend scheduled PrEP visits
- No contraindication to use of tenofovir disoproxil fumarate and lamivudine
- Bodyweight ≥30 kg
- Estimated glomerular filtration rate ≥60 mL/min (serum creatinine is recommended before PrEP initiation for individuals who are aged >50 years, have a history of hypertension and diabetes mellitus, have BMI <18.5 kg/m^2^, are receiving nephrotoxic medications, or have any symptoms or signs suggestive of renal impairment)
- No known renal diseases
- No diabetes mellitus

### Potential PrEP-User Participant Recruitment and Sample Size

#### Overview

We will enroll 250 PrEP-eligible individuals (aged ≥15 years) who are presenting to care at an urban STI clinic colocated on a district hospital campus in Lilongwe, Malawi. As a pilot study, no power calculations were pursued—sample size was determined based on expected recruitment feasibility. Among potential PrEP users, that is, individuals who are eligible for PrEP according to MOH guidelines [63], there are three subgroups: (1) PrEP users who are initiated on PrEP at their index STI clinic visit; (2) patients who are eligible for PrEP, but decline PrEP at their index STI clinic visit; and (3) referred partners from group 1 who are eligible for and agree to initiate PrEP. Demographic information will be collected from referred partners who decline or are ineligible for PrEP, but they will not be enrolled in the study. All participant recruitment occurs on-site at the STI clinic by trained study nurses. All groups will be enrolled simultaneously.

Given the objectives of this pilot study, we will intentionally recruit participants to represent a mix of age (15-24 years vs ≥25 years) and sex. Groups 1 and 3 (PrEP initiators) will comprise approximately 200 participants in total. We expect that approximately 60% (120/200) of index PrEP users enrolled will be women, reflecting the historic demographics of the STI clinic population. We will attempt to have approximately 30% (60/200) of index PrEP users enrolled aged between 15 and 24 years at enrollment. There are no historical estimates for partner eligibility or uptake among index patients who are HIV-negative (one of the objectives of this study), but we estimate that approximately 75% (150/200) of the PrEP-user participants will be from group 1.

#### Group 1—PrEP-User Participant (Approximately 150 Participants)

All patients in the STI clinic will receive a brief overview of the study design and objectives during the standard educational session provided to all individuals queuing for STI services. All individuals seeking STI services proceed first to HIV testing and counseling services (HTSs), according to Malawi standard of care. As described in Figure 1, patients eligible for PrEP based on HIV status are then referred to the on-site PrEP provider, who confirms PrEP eligibility and can also provide necessary STI screening and treatment. Then, potential study participants are referred to the on-site study nurse, who conducts additional screening for study eligibility. If eligible (Textbox 1), participants will be offered study enrollment and will complete an informed consent form. Eligible individuals who choose not to participate will be referred by the study nurse and proceed with any additional standard clinical management for syndromic STIs. Eligible participants may choose to defer enrollment for up to 7 days from the date of their index STI clinic visit. In this case, upon their next presentation to the clinic, they are referred directly to the study nurse, who reverifies eligibility and proceeds with informed consent.

Individuals who initiate PrEP at their enrollment visit are not under any obligation to continue PrEP throughout the study period and may choose to stop and start PrEP at their discretion.

#### Group 2—Individuals Who Declined PrEP (Approximately 50 Participants)

We expect that most PrEP-eligible individuals seeking STI services will decline PrEP. We will recruit approximately 20% (50/250) individuals who decline PrEP at their STI clinic visit into the prospective cohort. Of note, these individuals may choose to initiate PrEP later in the follow-up period, but will remain in group 2, regardless of subsequent PrEP use. Individuals who decline PrEP will be referred by HTS counselors to the study nurse.

#### Group 3—Referred Partners (Approximately 50 Participants)

Tracing procedures for named sexual partners (named by group-1 participants) are described in the following sections. Upon presenting to the clinic, partners will undergo screening for both study and PrEP eligibility by the study nurse. Interested partners will receive HIV testing services. If confirmed to be eligible for PrEP (ie, HIV-uninfected), they will be offered PrEP and enrollment into the study. Partners who are eligible for PrEP but decline it or those who are ineligible for PrEP will be dismissed from study participation. We expect that approximately 25% (50/200) of the PrEP-user participants will be from group 3; however, there is no formal cap for this enrollment. Enrolled partners will count toward the total enrollment goal of approximately 200 PrEP initiators (groups 1 and 3).

### Partner Elicitation (aPN)

#### Overview

We will follow the established WHO and President’s Emergency Plan for AIDS Relief protocols for eliciting sexual contacts [64,65] from individuals initiating PrEP; participants will be asked to provide the name and locator information for all sexual partners in the preceding 6 months. Participants will be asked to refer sexual partners to the clinic and will be provided with cards to distribute to their partners. Each card will request the recipient to report to the STI clinic with the card, and it will contain a number that links them back to the index participant. Counselors will encourage participants to distribute these cards to all individuals the participant has had sexual contact with in the preceding 6 months. In our surveys and in-depth interviews, we will explore preferences for four different aPN strategies:

1. Client referral—index clients will contact the partner and inform them that they should be screened or treated for STIs
2. Provider referral—the health care providers will contact the named partners directly and suggest they be screened for STIs, without telling them the index client’s name (ie, this will be done anonymously)
3. Contract referral—the index client can contact the partner within a certain period (typically 7-14 days), after which the provider will contact the named partner directly if they have not returned for screening, again without telling the index client’s name to the partner (ie, this will be done anonymously)
4. Dual referral—the provider can sit with the index client and their partner and support the client as they tell the partner about their STI

#### Sexual Partner Tracing

In accordance with the contract referral approach, if the named partners do not present to an STI clinic within 7 to 14 days, community outreach workers will use the tracing information (ie, phone number and address) to contact the partners and counsel them to visit the clinic. Contact may be made through telephone, SMS text message, or in person, as needed, and the name or identity of the index patient will not be disclosed.

Upon presentation to the clinic, sexual partners will be screened for PrEP eligibility and offered the opportunity to participate in the study. If they are eligible and agree to participate, they will receive the same enhanced PrEP services as index patients, including etiologic STI testing and partner referral. Partners who choose not to participate in the study will be offered STI screening and treatment services consistent with the standard of care for contacts of STI cases, including PrEP if interested and otherwise eligible. We will maintain a deidentified log of PrEP eligibility and PrEP uptake for partners who do not participate in the study.

### Data Collection

#### Overview

All study visits will be completed at the STI clinic, with study visits at enrollment and at 0, 1, 3, and 6 months aligning with the distribution of oral PrEP (Table 1). Group-2 participants will have visits at 0, 3, and 6 months.

**Table 1:** Schedule of events.

Evaluation	Baseline	Month		
		1^a^	3	6
PrEP^b^ eligibility screening	✓	N/A^c^	N/A	N/A
Rapid HIV antibody test	✓	✓	✓	✓
Mastercard^d^/PrEP refill review	N/A	✓	✓	✓
Syndromic STI^e^ assessment	✓	✓^f^	✓^f^	✓^f^
HIV RNA testing^g^	✓	+/−	+/−	+/−
STI testing (urine and blood)^h^	✓	N/A	✓	✓
Sexual partner elicitation	✓	✓	✓	✓
In-depth interviews^i^	✓	✓	✓	✓

^a^Month-1 visit is only for individuals who are initiated on PrEP at enrollment.

^b^PrEP: pre-exposure prophylaxis.

^c^N/A: not applicable.

^d^Mastercards are the paper-based medical record for each person who initiates PrEP at the clinic.

^e^STI: sexually transmitted infection.

^f^During follow-up visits, patients will be asked regarding any symptoms, and a physical examination will be conducted if symptoms are reported, with treatment provided according to presenting clinical symptoms and national guidelines.

^g^HIV RNA testing will be performed at baseline for all participants and at follow-up for anyone reinitiating PrEP, as defined by Malawi PrEP guidelines.

^h^Tests will be conducted for *Neisseria gonorrhea*, *Chlamydia trachomatis*, and syphilis (rapid plasma regain, with *Treponema pallidum* particle agglutination if rapid plasma regain titer is detectable) at each visit regardless of symptoms. If symptoms are reported by participants at the scheduled 1-month or patient-initiated interim study visit, specimens will also be collected for STI testing. Any infection detected by testing will be treated according to Malawi STI treatment guidelines.

^i^In-depth interviews may occur adjacent to any scheduled study visit.

#### Surveys

Surveys will be conducted among clinic staff and potential PrEP users via face-to-face interview. Clinic staff will respond to a Likert-scale survey evaluating the acceptability and feasibility of the integrated and enhanced PrEP strategies under investigation [62] at baseline and approximately 6-month follow-up visit (Multimedia Appendix 1).

Potential PrEP users (all groups) will respond to multiple tablet-based surveys at each visit, assessing perceived HIV risk, sexual behaviors (ie, partners and condom use), STI symptoms, PrEP adherence and side effects, PrEP refill (according to the documented clinic PrEP records), acceptability of PrEP receipt within the STI clinic setting, partner referral, and etiologic STI testing (Table 2). The visit timing aligns with the Malawi PrEP follow-up schedule [63].

**Table 2:** Patient and clinic staff participant survey content.

Outcome	Patient participants					Clinic staff	
	Baseline	1 month^a^	3 months	6 months	Baseline		6 months
Acceptability^b^	✓	N/A^c^	N/A	✓	✓		✓
Feasibility^b^	N/A	N/A	N/A	N/A	✓		✓
Appropriateness^b^	N/A	N/A	N/A	N/A	✓		✓
PrEP^d^ use^e^	✓	✓	✓	✓	N/A		N/A
Contraception^f^	✓	✓	✓	✓	N/A		N/A
Perceived HIV risk	✓		✓	✓	N/A		N/A
Reasons for initiating (or declining) PrEP	✓	N/A		✓	N/A		N/A
Number of sexual partners^g^	✓	N/A	✓	✓	N/A		N/A
Condom use	✓	N/A	✓	✓	N/A		N/A
HIV status of partners	✓	N/A	✓	✓	N/A		N/A

^a^There is no month-1 visit for group-2 participants.

^b^Refers to acceptability, feasibility, and appropriateness specific to etiologic sexually transmitted infection testing, assisted partner notification, and integration of sexually transmitted infection and pre-exposure prophylaxis services.

^c^N/A: not applicable.

^d^PrEP: pre-exposure prophylaxis.

^e^Adherence to PrEP is asked to group-1 and group-3 participants and any group-2 participant who decides to initiate PrEP during the follow-up period. Reasons for discontinuation is asked to individuals with gaps in use or intended cessation of use.

^f^Asked only to female participants.

^g^At baseline, participants are asked about partners in previous 6 months; during follow-up visits, participants are queried about the number of partners since their previous visit (regardless of interval) and in the past month.

#### Individual In-depth Interviews

The content of the in-depth interview guides will be informed by Conceptual Model for Implementation Research by Proctor [66]. Clinic staff in-depth interviews will focus on experience with prescribing PrEP (ie, structural or clinic-specific challenges to distribution), experiences with eliciting partners for PrEP referral (ie, refusals, index preferences for provider-initiated referral, contract referral, or dual referral), perceptions regarding how PrEP is being used (ie, perceived patient adherence to PrEP, fluctuations in PrEP use, or barriers to ongoing engagement in PrEP care), perceptions regarding alternative PrEP formulations (eg, injectable and insertable), approach to HIV risk assessment, and communication strategies regarding risk. To better elucidate the possible pathways through which STI testing may facilitate PrEP persistence, we will explore how or if the presence of an STI in the PrEP user influences counseling. As clinicians and clinic staff are the primary implementing actors for the integrated STI+PrEP strategy, we will also evaluate the perceived barriers to or facilitators of provision of this new clinical service. Refer to Multimedia Appendices 1 and 2 for sample in-depth interview guides.

A subset of approximately 25 potential PrEP users will be recruited to participate in the in-depth interviews. The final number and distribution of interviews between each PrEP user group will depend on thematic saturation based on real-time review of transcripts. Topics include motivation for starting or stopping PrEP, perceived HIV risk, and perceived barriers to PrEP use, specifically when managed through an STI clinic. Probes will be determined based on whether respondents accepted or refused PrEP initially. For example, among PrEP acceptors, we will elicit responses regarding how perceived risk influences PrEP use, thus providing additional depth to the survey questions. Interviews will also explore how or if the presence of a symptomatic or asymptomatic STI may influence their HIV risk perception and perceived need for PrEP. Finally, we will examine the preferences for nonoral PrEP formulations, including LAI PrEP, with all patient participants.

#### Clinic Record Review

We will extract data from existing clinic records to examine clinic-wide uptake and PrEP persistence. Source documents include an electronic medical record that captures basic demographics (age and sex) and critical clinical outcomes (HIV status) for each clinic encounter at the STI clinic; the PrEP register, which documents all individuals who are offered PrEP within the clinic and captures basic demographics (age, sex, and pregnancy status); and PrEP *mastercards*. Mastercards are the paper-based medical record for each person who initiates PrEP at the clinic; the document captures HIV risk (Textbox 2) and includes a PrEP readiness assessment documenting additional PrEP eligibility and a table corresponding to each PrEP visit in which HIV-negative serostatus is confirmed and STI screening, if done, is documented.

#### Anonymous Survey Among Individuals Not on PrEP

A convenience sample of individuals who are not initiated on PrEP will be asked to respond to a brief survey capturing age, sex, whether they were offered PrEP, and reason for declining PrEP. The purpose of this survey is to help estimate the proportion of patients in the STI clinic who were not enrolled in the study but were offered PrEP, and among those offered PrEP, to understand the reasons for declining. Patients will be approached as they are checking out at the reception desk and asked to respond to the brief survey. Patients who are enrolled into the study will not be surveyed. Among individuals who are not offered PrEP, we will also record HIV status from the same-day HTS.

#### Laboratory Evaluations

According to the Malawian guidelines, all patients with STI at Bwaila STI undergo HIV testing via fingerstick blood collection using 2 rapid test protocols, unless a person has documentation of HIV infection [63]. Participants with newly diagnosed HIV infection or those who are not on antiretroviral therapy (ART) will be referred to the colocated HIV clinic for management.

Before PrEP initiation, patients will undergo any necessary screening tests to confirm the safety of PrEP, as provided by MOH PrEP programs. At the time of submission, same-day PrEP initiation is recommended, with discontinuation if baseline estimated glomerular filtration rate is <60 mL/min. Currently, hepatitis B testing is part of PrEP initiation tests offered through MOH; however, stockouts sometimes preclude testing; results obtained from rapid hepatitis B testing, if conducted, will be entered into appropriate study case report forms. Consenting patient participants in all 3 groups will undergo STI testing at baseline and follow-up visits (Table 1). Urine specimens will be tested for *Chlamydia trachomatis* and *Neisseria gonorrhoeae* using Xpert CT/NG cartridge and GeneXpert System platform (Cepheid). Syphilis rapid plasma regain (RPR) titer (BD Macro-VUE; Becton, Dickinson and Company), with confirmatory *Treponema pallidum* particle agglutination (Serodia Fujirebio Inc), if RPR is positive, will be analyzed on blood specimens. Repeat RPR or *Treponema pallidum* particle agglutination will be conducted if the previous test was nonreactive at 3 months or if signs or symptoms of new syphilis infection are present. Repeat RPR titer will be conducted regardless of baseline RPR at the 6-month visit. Individuals initiating PrEP will be screened for acute HIV infection with HIV RNA testing using GeneXpert viral load cartridge (Cepheid). Anyone with detectable HIV RNA will be contacted immediately, and PrEP will be discontinued with prompt ART referral.

### Study Outcomes

Our primary outcomes evaluate the acceptability, feasibility, and effectiveness of (1) PrEP integration into STI services and (2) the enhanced PrEP package (aPN and STI testing).

Acceptability and feasibility of PrEP integration into STI services is measured using patient and clinic staff surveys, contextualized by determinants of integration, as examined using qualitative interviews [66]. Effectiveness is defined in terms of PrEP uptake and persistence at 1, 3, and 6 months. We will examine PrEP initiations as a proportion of all STI clinic patient visits and reasons for declining PrEP among individuals eligible but not starting PrEP. Among individuals who initially refuse PrEP, we will describe how many subsequently initiate PrEP. We will assess PrEP persistence according to clinic attendance, PrEP refills abstracted from mastercards, and self-reported PrEP adherence in study surveys, evaluating the frequency of appropriate PrEP refills and engagement with care. We will also evaluate PrEP persistence in the general clinic population using clinic record review among individuals who started on PrEP during the study recruitment period but who were not enrolled in the study. Finally, we will examine the reasons for PrEP discontinuation among individuals who terminate use during the study follow-up period.

Acceptability and feasibility of aPN as part of PrEP care is similarly assessed with patient and clinic staff surveys and qualitative interviews, examining preferences for aPN strategies from multiple perspectives. Effectiveness of the aPN strategy as a component of PrEP care is evaluated using partner referral outcomes and PrEP uptake among eligible partners. Specifically, referred partner metrics will include the following: number of partners referred per index, proportion of index participants naming ≥1 recent sexual partner, proportion who have ≥1 recent sexual partner returning to the clinic (with or without community tracing), and proportion of all returning partners who are eligible for and initiated on PrEP. To explore strategies that may enhance or improve aPN, we will describe participant preferences regarding the sex, age, and cadre (ie, health professional vs peer) of the person eliciting partners for naming and subsequent tracing.

Finally, acceptability and feasibility of etiologic STI testing is examined using patient and clinic staff surveys and qualitative interviews, exploring perceived barriers to and facilitators of integrating testing into usual care. Effectiveness of this strategy as a component of PrEP care will examine incident infections (including asymptomatic infections that would be otherwise missed by syndromic management among index and partner participants) and proportion of participants receiving appropriate STI treatment within 7 days of testing. An incident infection is defined as a new positive diagnosis based on follow-up testing (Table 1). If a patient had a positive result at their previous visit, we will distinguish between persistent versus incident infections based on whether the participant had received appropriate pathogen-directed treatment for their previous infection. All STI treatment will be offered in concordance with local management guidelines.

Secondary outcomes include fluctuations in HIV risk, including reports of unprotected encounters with sexual partners with unknown HIV infection status or known infection not on ART, multiple sexual partners without consistent condom use, and sexual partners who may have other partners. We ask patients to report any primary or casual partners in the month before the interview, and thus assess the rates of concurrency. Furthermore, by having primary partners *named* within our survey tool, we are able to query regarding any changes in primary partners during the 6-month follow-up period. Changes in HIV risk will be evaluated alongside reported perceived risk of HIV (assessed at each follow-up visit). We will examine partnership patterns within the cohort, including the proportion of participants who report no change or new partners throughout the follow-up period, and characterize partner switching among those with multiple partners, including primary and casual partners. Finally, we will evaluate predictors of PrEP persistence at each time point (1, 3, and 6 months) and those factors that are associated with PrEP discontinuation.

### Planned Statistical Analysis

Planned analyses are primarily descriptive in nature, summarizing participant characteristics, behaviors, and perceptions. We will examine differences between nonreferred participants who initiated PrEP (group 1) and those who did not initiate PrEP (group 3), with a particular focus on HIV risk behaviors, PrEP perceptions, and self-perceived HIV risk. Exact test by Fisher will be used to compare the differences in proportions between arms for categorical variables, and *t* tests will be used for continuous data (a=.05).

We will additionally evaluate longitudinal changes in HIV risk behaviors, perceived HIV risk, and PrEP use, both at the individual and population levels. To examine individual-level behavior changes, we will characterize each participant’s behaviors and perceptions over the course of follow-up and compare patterns by PrEP initiation group. To examine population-level behavior changes, we will calculate predicted probabilities and 95% CIs for our behaviors of interest by visit, using generalized estimating equations to account for within-participant correlation. Both individual-level and population-level analyses will focus on partner type (primary or steady vs casual) and incident STIs, with comparisons among participant groups (group 1, group 2, and group 3).

We will explore risk factors associated with PrEP discontinuation using logistic regression among participants who start PrEP at enrollment or during the study follow-up period.

### Planned Qualitative Analysis

For qualitative work, interviews will be transcribed in English from either English or local language audio recordings. Transcripts from users and clinic staff will be analyzed separately and reviewed for quality. Interview transcripts and field notes will be thematically analyzed using a combination of deductive and inductive analytic approaches. An initial codebook will be developed based on a priori concepts driven by the theoretical underpinnings used to develop the semistructured questionnaire, specifically the Conceptual Model for Implementation Research by Proctor [66]. Then, all textual data will be read thoroughly to summarize first impressions. Emerging themes will be incorporated into the codebook. Pre-existing codes may be modified based on interview transcripts. Transcripts will be coded iteratively in qualitative computer software programs. Researchers will code interviews separately to assess intercoder reliability. The codebook will be revised and updated. Analysis of the coded data will include investigation of relationships among codes, coding of matrices, and mapping of codes and themes. Identifying any hierarchical structure among themes also helps to determine how the codes fit together. Themes may be compared across groups by triangulating data.

### Ethics Approval

This study has been approved by the University of North Carolina at Chapel Hill Biomedical Institutional Review Board (21-2457) and Malawi National Health Services Research Committee (21/09/2777). Potential participants will be provided up-to-date information, and they will provide consent before any study procedures. All research procedures will adhere to Malawi and US ethical standards for research involving human participants.

Malawi guidelines allow those aged ≥15 years to initiate PrEP if they meet other eligibility requirements. In the proposed study, we independently consent PrEP users aged between 15 and 17 years, without additional separate parental consent based on their legal right and eligibility to receive PrEP, the minimal risk posed by participating in the described study, and the potential for adolescents to directly benefit from the outcomes of this study. We provide adequate protection regarding the confidentiality of all study activities, including receipt of care, participation in interviews, and referrals for services, if needed. The study has been registered in ClinicalTrials.gov (NCT05307991).

## Results

Enrollment began in March 2022 and is projected to continue until February 2023, with patient participant follow-up through August 2023. As of November 14, 2022, we have enrolled 78.8% (197/250) of the patient participants (group 1: 146/197, 74.1%; group 2: 29/197, 14.7%; and group 3: 22/197, 11.2%). The results of this study are expected to be reported in 2024.

## Discussion

### Overview

This study examines the acceptability, feasibility, and effectiveness of an enhanced PrEP package as part of a PrEP implementation strategy that integrates PrEP services with STI care in Lilongwe, Malawi. The efficiency and effectiveness of integrated services are at the core of this pilot study, leveraging the existing infrastructure and staff to recruit individuals already presenting for STI care. As PrEP availability expands around the world, including in SSA, novel delivery strategies are needed to recruit and retain the individuals at highest risk of acquiring HIV.

Linking individuals seeking care for STIs to PrEP is a logical approach to integrate complementary sexual health services [67-69]. Individuals seeking care at STI clinics are not only at increased risk of HIV but also almost universally eligible for PrEP under many national guidelines [70]; identifying eligible PrEP users at STI clinics may reach those with limited contact with the health care system [68,69,71-74]. In the United States, urban demonstration projects show high interest when PrEP is offered in STI clinics [75,76], with good uptake and adherence [77], and this integrated strategy may be more effective for reducing HIV incidence compared with community-based PrEP recruitment [78]. Our study fills a critical knowledge gap in SSA regarding the acceptability and feasibility from both patient and clinic staff perspectives of what has been shown to be a promising integrated PrEP and STI implementation strategy elsewhere.

Our study expands on the integration of complementary services (PrEP and STI) by examining a novel application of an evidence-based intervention (aPN). A reliable workhorse for finding individuals previously unaware of HIV infection and linking them to ART, to the best of our knowledge, aPN has not previously been used as a strategy to identify potential high-risk PrEP beneficiaries besides individuals linked to PrEP based on sexual partners who have been newly diagnosed with HIV [31]. Our surveys collect detailed information regarding partnership patterns and associated HIV risk, characterizing partner switching, awareness of partner HIV status, and, as relevant, awareness of HIV treatment among HIV-infected partners. Coupled with our partner notification approach and insights regarding returning partners’ HIV status and PrEP uptake, we will be uniquely positioned to explore dynamics of partner stability and HIV risk that may influence PrEP use.

Linkage of partners to PrEP and STI testing services may be particularly relevant when coupled with etiologic STI testing. Hardly a novel part of PrEP care in some parts of the world, evaluating the frequency of asymptomatic STIs among both PrEP users and their sexual partners could have important implications for STI management in much of SSA, where most countries continue to rely on less sensitive and less specific syndromic management. We are also exploring how or if PrEP counselors integrate STI test results into PrEP counseling and whether an STI diagnosis, with or without symptoms, influences perceived risk of HIV for individuals on PrEP. These more nuanced features of attitudes toward STI testing and their potential influence on PrEP use will be examined qualitatively.

Although 6 months of follow-up likely only captures a brief portion of the full *at-risk* period for PrEP initiators, this study will provide unique insights into fluctuations of HIV risk behaviors, perceived HIV risk, and PrEP use—including starting and stopping PrEP. Understanding the interplay between these 3 factors may help to inform PrEP distribution and counseling strategies in the setting of shifting risk behaviors. By prospectively monitoring changes in HIV risk, including biomarkers of risk (ie, incident STI), among a cohort of individuals seeking STI services, our outcomes may help to inform the development of *prevention-effective* PrEP use outcomes and programmatic objectives [79,80].

PrEP persistence is best considered in the context of contemporaneous HIV risk behaviors. Poor PrEP persistence, including suboptimal adherence [20,32] and discontinuation [33-40], threatens PrEP effectiveness when interruptions occur during periods of high HIV risk. In contrast, if PrEP persists in times of low HIV risk, users may experience unnecessary side effects, and scarce resources may be misallocated. The concept of PrEP use relative to sexual risk has been described previously [79], but few studies have considered the concept of alignment between PrEP use and risk perception or behavior in describing or interpreting PrEP persistence outside known serodiscordant partnerships [81]. Outcomes from this pilot study lay the foundation for a better understanding of these complex behavioral patterns that are at the core of defining and supporting appropriate and effective PrEP use.

### Limitations

There are potential challenges associated with this pilot study; our observational design may limit conclusions regarding causality of, for example, etiologic STI testing and PrEP persistence. The retention of participants is likely enhanced as they are being provided transport incentive to participate in study activities and are traced via phone or in person for missed study visits. These efforts artificially increase retention in the study; however, ongoing PrEP use is not a requirement for participation. We are addressing this by comparing PrEP refill records and routine metrics of PrEP use among study participants and nonparticipants receiving PrEP at Bwaila during the study enrollment period. Our measurement of PrEP use is imperfect, relying on refill timing and self-reported use. This may overestimate or underestimate true adherence, and future studies may seek more objective measurements of PrEP exposure using urine, blood, or hair.

Another challenge is accurately capturing PrEP uptake at the clinic level, as PrEP may not be offered to all individuals who are eligible. We are approaching this challenge in two ways: first, we are collecting exit interviews for the first approximately 3 months of study enrollment, in which we will examine the proportion of patients in a convenience sample who self-report being offered PrEP; second, we are discussing PrEP eligibility, integrated services, and attitudes toward PrEP referral with clinic staff during qualitative interviews. We believe that these discussions can help to contextualize observed referral *rates* and inform future investigations that may seek to increase PrEP referrals.

There is potential measurement error surrounding our incident STI outcome, specifically if individuals seek treatment elsewhere for symptoms of an STI during the study follow-up period. We have tried to mitigate this by requesting patients to return to the clinic for any concerning symptoms. Our assessment of STIs is not exhaustive, with the notable omission of testing for trichomoniasis and herpes simplex virus. Physical examinations would help identify patients with any concomitant symptoms, and we will use these estimates in a sensitivity analysis to examine potential missed diagnoses. However, as we know that syndromic diagnosis is relatively insensitive (missing individuals who are asymptomatic) and, particularly for nonulcerative disease, not very specific, we may be underestimating incident STIs within this population.

Injectable PrEP, which requires every 8-week dosing rather than daily oral pills, is an exciting development, but may have different determinants of uptake within the STI clinic and among the clinic population. If LAI PrEP becomes approved for use in Malawi during the study period, this formulation can be offered to eligible participants, consistent with MOH eligibility and recommendations. Although the intention of this study is not to directly compare LAI with oral (daily) PrEP, our study design and the content of our in-depth interviews directly acknowledge this forthcoming technology, and we are equipped to examine relevant PrEP uptake and persistence outcomes for both oral *or* LAI.

### Conclusions

Despite these limitations, this study lays a foundation for future large-scale evaluations of an integrated PrEP strategy—informing the feasibility of otherwise resource-intensive interventions such as partner tracing and STI testing. These preliminary data can be used to plan PrEP programs that address challenges in optimizing PrEP across the PrEP cascade—from identifying the appropriate candidates for PrEP to retaining these individuals in a *risk*-*aligned* manner, such that the need for PrEP and counseling on benefit of PrEP reflect HIV risk.

## Appendix

Multimedia Appendix 1 Clinic staff in-depth interview guide.

Multimedia Appendix 2 Patient in-depth interview guide.

## Abbreviations

aPN: assisted partner notification
ART: antiretroviral therapy
HTS: HIV testing and counseling service
LAI: long-acting injectable
MOH: Ministry of Health
PrEP: pre-exposure prophylaxis
REDCap: Research Electronic Data Capture
RPR: rapid plasma regain
SSA: sub-Saharan Africa
STI: sexually transmitted infection
UNC: University of North Carolina
WHO: World Health Organization

## Data Availability

The data set anticipated to be generated during this study will be available from the corresponding author upon reasonable request.
