# Supplementary material for: Integrating Enhanced HIV Pre-exposure Prophylaxis Into a Sexually Transmitted Infection Clinic in Lilongwe: Protocol for a Prospective Cohort Study
Source: JMIR Res Protoc. 2022 Dec 5;11(12):e37395. doi: 10.2196/37395 (PMC9764156; doi:10.2196/37395)
Supplement: Multimedia Appendix 1 [file resprot_v11i12e37395_app1.docx]

**Supplemental Materials: Example clinic staff interview guide**

*[Read or paraphrase to participant]*

Thank you for agreeing to talk with me today. My name is [NAME]. I am part of a research team seeking to improve HIV prevention in Malawi, specifically through use of pre-exposure prophylaxis. As you may know, pre-exposure prophylaxis (or PrEP) can help reduce the risk of an HIV-uninfected person becoming infected with HIV, if the PrEP medication is taken as prescribed.

We want your honest opinions – there are no right or wrong answers, what we want to know is what you think and believe. If there are any questions that make you feel uncomfortable, feel free to tell me and we can skip or come back to those questions.

Anything that you say to me will be kept confidential. If you have consented to audio recording, our discussion will be audio recorded and then transcribed. If you did not consent to audio recording, we will take notes to capture responses. When it is written out, all information that could identify you to someone reading the words (anything said with names or locations or that kind of thing) are completely removed. You may opt not to have the interview recorded if you wish. The material from our interview is saved with your identification number but not your name. The research team members reviewing this text will not link who you are to that ID and will not tell anyone on site about your specific feedback. There are other people doing interviews as well, and we will put all those documents together and look at main topics people talked about. We will not identify or share anything you say as a comment linked to your name or ID number.

***Introduction***

To start off, I’d like to learn a little more about your professional role.

1. Can you please tell me about your role here at the clinic?

*Probes:* Walk me through a day at work.

Who do you interact with on a daily basis? (HINT: other staff, supervisors, clients)

What do your interactions with other staff look like? How do you work together?

What are your main responsibilities?

How long have you been in this position?

***PrEP and aPN knowledge/experience***

1. Could you begin by telling me if you had ever heard of pre-exposure prophylaxis (PrEP) for HIV prevention before this interview, and what you know about it?

*Probes:* If you have heard about it but don’t know what it is, what have you heard about it?

1. Can you tell me how you first learned about PrEP?

*Probes:* Had you ever heard of PrEP before being approached for this study?

What information did you receive about PrEP and from what source?

What did you think about PrEP when you first learned of it?

1. What kind of experience do you have providing, prescribing, or counseling patients on use of PrEP?
2. Besides PrEP, what HIV prevention methods do you counsel your patients on?

*Probes:* Do you find that your patients are typically receptive to these prevention options?

***Integrating PrEP and existing STI clinical services***

Thank you for sharing with me. Next, I’d like to ask you a little more about PrEP and some of the different ways it may be delivered or incorporated into existing clinical services

1. In general, who do you think could benefit from PrEP?

*Probes:* What sort of patient demographics?
Are there people that should NOT take PrEP?
What are some of the ways you would think about counseling someone on how to decide if PrEP was the right decision for them?
Do you think persons who are coming to the STI clinic are at risk of HIV? How would you compare their risk to other so-called “key populations”? Do you believe these persons perceive themselves to be at risk of HIV? Why or why not?

1. How or where is PrEP currently available for persons at risk of HIV?

*Probes:* What sort of places do you think would be appropriate to offer PrEP? Why?

1. Have you had persons ask you for PrEP when they are seeking STI treatment? If so, where have you referred them or what have you told them?
2. What kind of barriers or challenges do you foresee if PrEP distribution were to be integrated into existing STI clinic services at Bwaila?

*Probes:* Any concern about space? Personnel? Other resources?

What about for PrEP follow-up visits and willingness to return to STI clinic for follow-up care?

1. What do you perceive to be some of the benefits of incorporating PrEP distribution into existing STI clinic services at Bwaila?

*Probes:* Any concern about space? Personnel? Other resources?

What about for PrEP follow-up visits?

1. What other clinical or non-clinical venues do you feel may be appropriate for distributing PrEP?

*Probes:* How important is it that a person receiving PrEP interact with a clinician when they first start PrEP? What about during their regular PrEP follow-up visits?

1. Have you heard about different types of PrEP? That is, alternative to a daily oral pill?

*Probes:* What do you feel may be some of the benefits of alternative PrEP formulations?

What may be some challenges? Any specific benefits or challenges were these alternative formulations to be integrated into STI care?

I would like to talk to you specifically about injectable PrEP. In this kind of PrEP, instead of taking pills every day, patients would receive injections once per month at the clinic. The injection would include similar drugs to what is in the oral pill, and they would remain in the patient’s body, helping to protect from HIV infection, for an entire month. They would likely need to come back to the clinic once per month to get the injections. These drugs have been studied in people without HIV for HIV prevention and were safe and effective. Once a person starts receiving the injections it is important that they come to the clinic every month, but, if they decide they don’t want to continue with the injections, they can go back to the daily pills. Injectable PrEP is not yet available for HIV prevention in most countries but may become available soon.

1. Have you ever heard of this type of PrEP before?

*Probes:* Where did you hear about it?
What sorts of things have you heard about it?

1. Based on what we’ve just discussed, what is your initial reaction to monthly injectable PrEP?

*Probes*: Can you tell me anything that is appealing to you about it?

What questions or concerns do you have about it?

1. Imagine your clinic were to offer the option of daily oral or monthly injectable PrEP to your clients. How would you, personally, feel about this change?

*Probes*: How would you decide when to offer daily oral? Long acting injectable?

How would you feel about clients having the option of choosing?

What kinds of clients might injectable PrEP be most appropriate for?
 What kinds of challenges for clients might injectable PrEP address?

1. When you imagine incorporating monthly injectable PrEP into the clinic’s offerings, what might be some challenges for your clinic as a whole?

*Probes:* What kinds of changes would need to happen in the clinic for the transition to be successful? (HINT: new protocols, training staff, changing roles, space/facilities)

Can you think of any other times your clinic started a new medicine or technology? What kinds of changes happened and how did you respond?

What do you think your colleagues would think about monthly PrEP?

1. When you imagine incorporating monthly injectable PrEP into your own daily practice, what might be some professional challenges you face as a (clinician/counselor/pharmacist/clinic director)?

*Probes*: In what ways would your job be easier?

In what ways would your job be more difficult?

How do you imagine your day-to-day job might change?

What are your thoughts about…

…Patient visits increasing in frequency?

…Adjusting to new procedures (e.g., training staff on injecting)?

…Ordering or storing new medications and supplies?

…Counseling patients on new side effects (e.g., injection site swelling)?

1. Thinking specifically about your clients currently on PrEP, how might they feel about monthly injectable as an alternative to the daily oral tablet?

*Probes:* How appealing do you think it would be to your clients?

How willing would the average client be to switch?

What might they be hesitant about?

How would you counsel them?

1. Now, let’s continue thinking about patients you take care of. Imagine a new client comes into the clinic who has never used PrEP, or who used it in the past but stopped. How do you think they might feel if you told them about monthly injectable PrEP?

*Probes:* How attractive would a monthly injection be to a new client when compared with a daily oral tablet?

1. In what kind of setting do you think these injections should be offered?

*Probes:* For example, do you think people would want to come back to the STI clinic? Have the injection administered in the community? Some other clinical setting?
Would you feel it appropriate to have a non-clinician giving this injection?

1. Thank you for your answers so far. I have one final question. Can you tell me one thing that excites you and one thing that concerns you about incorporating injectable PrEP into your clinic?

***Enhancing PrEP using aPN and etiologic STI testing***

Thank you for sharing with me. Next, I’d like to ask you a little more about some possible strategies to improve PrEP uptake and possibly persistence on PrEP. Again, we would like to hear your honest opinions and perceptions – there are no right or wrong answers.

1. What kind of experience do you have providing, prescribing, or counseling patients on partner notification for sexually transmitted infections or HIV?

*Probes:* What have you found is particularly effective when eliciting partners from patients with a new STI or HIV?

What are some of the challenges eliciting partners?

How successful do you feel assisted partner notification strategies are for helping bring back potentially exposed sexual partners for testing and possible treatment? How is tracing typically done and by whom? Do you have experience tracing partners or is there a system in place to notify/trace named partners and bring them into care? How much time does aPN take?

1. What do you feel may be some barriers to identifying potential eligible PrEP users using assisted partner notification, asking STI clinic patients to provide names/contact information for recent sexual partners and then contacting the partners to offer PrEP?

*Probes:* What sort of resources would something like this require? Do you think persons with an STI (without HIV) would be willing to name partners? What about persons with HIV (newly diagnosed or known positives)?

1. What do you fell may be some of the benefits of incorporating assisted partner notification into PrEP delivery at the STI clinic?

*Probes:* what do you find appealing about incorporating assisted partner notification as part of PrEP care? What are ways that assisted partner notification may be made more attractive or effective as part of PrEP care?

I’m going to now focus on testing for STIs in the context of PrEP delivery.

1. What proportion of all persons with STIs have symptoms? (symptoms could include discharge, belly pain, itching, sores, ulcers, etc)

*Probes:* do you think most people seek care when they have a symptom that may be an STI? Why or why not? Do they usually come to the clinic first or have they sought care elsewhere prior to coming to the clinic? What sort of places do they seek care first? What barriers, if any, do you feel people have about coming to an STI clinic?

1. What experience do you have with testing for STIs, other than for HIV? Examples might include hepatitis B or syphilis

*Probes:* In what context did you conduct these tests? Was it easy or different to interpret results? How did having these results influence your counseling for the patients or your perception of their risk?

1. What do you feel some of the barriers to integrating STI testing, with both urine and blood draws, might be if included as part of PrEP programs/follow-up visits?

*Probes:* Do you think most patients would accept testing? Why or why not?
What about return or results if the test was not back on the same day?
Do you think patients would perceive a benefit to having testing even if they did not have symptoms?
What about “believing” the results?
What additional resources would be needed in the clinic to facilitate testing (other than the actual tests, which would be done in the laboratory)?

1. What do you feel some of the advantages or benefits of integrating STI testing into PrEP care might be?

*Probes:* what do you find appealing about having STI testing as part of PrEP care?

1. In what ways would STI testing influence your counseling?
2. In what ways do you think STI testing might influence how you perceive the risk of the patient you are counseling?

*Probes:* in what way might STI testing influence the perceived risk of the clinic patient?

1. In what ways do you think STI testing may impact PrEP uptake or persistence on PrEP? When I say persistence, I mean continuing to stay on PrEP as prescribed
2. In what ways would STI testing influence how you counsel a person regarding their ongoing need for PrEP?

*Probes:* How do you feel you would counsel someone who says they do not need PrEP because they do not have any new STIs? How do you think you would know when someone is safe to stop PrEP and how would you counsel them regarding stopping PrEP?

***Closing interview***

Those are all of the questions that I have. Is there anything else you would like to share about your decision to use PrEP or your experience using it that we have not discussed?

Thank you for taking the time to talk with me. What questions do you have for me?
