# Supplementary material for: Integrating Enhanced HIV Pre-exposure Prophylaxis Into a Sexually Transmitted Infection Clinic in Lilongwe: Protocol for a Prospective Cohort Study
Source: JMIR Res Protoc. 2022 Dec 5;11(12):e37395. doi: 10.2196/37395 (PMC9764156; doi:10.2196/37395)
Supplement: Multimedia Appendix 2 [file resprot_v11i12e37395_app2.docx]

**Supplemental Material: Example patient interview guide**

*[Read or paraphrase to participant]*

Thank you for agreeing to talk with me today. My name is [NAME]. I am part of a research team seeking to improve HIV prevention in Malawi, specifically through use of pre-exposure prophylaxis. As you may know, pre-exposure prophylaxis (or PrEP) can help reduce the risk of an HIV-uninfected person becoming infected with HIV, if the PrEP medication is taken as prescribed.

We want your honest opinions – there are no right or wrong answers, what we want to know is what you think and believe. If there are any questions that make you feel uncomfortable, feel free to tell me and we can skip or come back to those questions.

Anything that you say to me will be kept confidential. If you have consented to audio recording, our discussion will be audio recorded and then transcribed. If you did not consent to audio recording, we will take notes to capture responses. When it is written out, all information that could identify you to someone reading the words (anything said with names or locations or that kind of thing) are completely removed. You may opt not to have the interview recorded if you wish. The material from our interview is saved with your identification number but not your name. The research team members reviewing this text will not link who you are to that ID and will not tell anyone on site about your specific feedback. There are other people doing interviews as well, and we will put all those documents together and look at main topics people talked about. We will not identify or share anything you say as a comment linked to your name or ID number.

***PrEP knowledge and decision***

I’d like to start off by talking about HIV pre-exposure prophylaxis, and your decision about whether or not PrEP was right for you.

1. Could you begin by telling me if you had ever heard of pre-exposure prophylaxis (PrEP) for HIV prevention before this interview, and if so, what you know about it?

*Probes:*

If you have heard about it but don’t know what it is, what have you heard about it?

Can you begin by telling me how you first learned about PrEP?

Had you ever heard of PrEP before being approached for this study?

What information did you receive about PrEP and from what source?

What did you think about PrEP when you first learned of it?

1. How did you make the decision to use or not use PrEP?

*Probes:*

Have you ever considered using PrEP before this study?

What prompted you to consider it?

Can you describe the decision process?

How was your partner(s) involved in this decision?

How would you have preferred your partner be involved in the decision?

Other than your partner/husband, how was your family involved in this decision?

How were clinicians or study staff involved in this decision?

Is there any one else you would have liked to have been involved in this decision or helping to discuss PrEP with?

1. (if deferring PrEP) Do you think PrEP is something you would ever consider using? What are the reasons for that? In what situations or times in your life would you consider using PrEP?
2. (if accepting PrEP) What motivated you to start PrEP? What made it appealing?
3. Were there any concerns that you had when you were deciding whether or not to use PrEP?

*Probes:*

How did your partner influence these concerns, if at all?

How did your family influence these concerns, if at all?

How did clinicians or study staff influence these concerns, if at all?

What things appeal to you about using PrEP?

What are some concerns you would have about using PrEP?

1. (if accepting PrEP) Since starting PrEP, what sort of challenges have you had remembering to take the medication?

*Probes:*

What sorts of strategies have you tried to remember to take the medication? How have these worked for you?

1. (if accepting PrEP) Have you considered or decided to stop taking PrEP all together? Can you think of a reason that you may stop taking PrEP?

*Probes:*

(if stopped PrEP) What were the reasons you decided to stop PrEP?

(if considered stopping PrEP) What were the reasons you considered stopping PrEP?

If you have stopped (or were to stop) PrEP, what, if anything, would make you consider restarting PrEP? Where would you go to restart PrEP and why would you go there?

(if considering stopping PrEP or not considering stopping PrEP) Have you thought about how long you plan to use PrEP? How will you make the decision to stop using PrEP?

1. Besides PrEP, what HIV prevention methods have you used or considered using?
2. Compared to those other methods you mentioned, would you prefer to use PrEP instead of or in combination with these other methods? What are the reasons for that?
3. Would you tell your partner that you were using PrEP?

***Perceived HIV risk***

1. How would you describe your risk of acquiring HIV?

*Probes:*

Why do you think that?

What are some of the ways you think you may be at risk for HIV?

What types of things do you do to protect yourself against that risk?

1. Does having an STI, or symptoms of an STI, change your perceived risk in any way? Why or why not?

*Probes:*

What about if you were told that you had an STI based on a urine or blood test but were without any symptoms?

1. Do you think taking PrEP would change your risk of getting HIV? Why or why not?

***PrEP and STI Care***

1. Prior to today’s visit, have you ever sought care for any sexually transmitted infections?

*Probes:*

Where have you previously sought this care? STI clinic? Pharmacy? Family planning? Somewhere else?

1. How do you feel about receiving your PrEP care through the STI clinic?

*Probes:*

What are some of the possible benefits of having your PrEP care co-located within the STI clinic?

What if any concerns do you have about receiving your PrEP care at the STI clinic?

1. Is there somewhere else you would prefer to receive PrEP follow-up?

*Probes:* For example, would you prefer someone came to your community? At a family planning clinic? Some other gathering place (ask for examples)?
What might be some of the advantages of receiving PrEP follow-up care outside of a clinical setting? What might be some of the disadvantages?

***aPN***

Thank you for your responses so far. Next, I would like to talk about something called assisted partner notification. This is a strategy to help find people who may benefit from STI or HIV screening and bring them in to the clinic.

1. Are you familiar with the idea of confidential partner notification for communicable diseases, like some STIs and HIV?

*Probes:*

Do you have any personal experience?

Any friends who you know who have experience with assisted partner notification?

*Offer clarification/reinforce knowledge regarding alternative aPN strategies*

1. Have you ever been notified by a healthcare worker that you may have been exposed to an STI or HIV and so should be tested?
2. How do you feel about giving a healthcare worker information (name, phone number, location) about your recent sexual partners, with the understanding that they may reach out to them to suggest that they come to the STI clinic?

*Probes:*Do you believe this can be done without compromising your identify?
What concerns do you have?
Are there some kinds of partners you would feel more comfortable given names/contact information for? Why or why not?

1. How do you think you would feel if you were contacted?
2. There are 4 different types of “partner notification” including: 1) provider referral, in which a counselor or other health care provider will call or visit your partner and offer them STI testing/screening; 2) client referral, in which you tell your partner about you results and encourage him or her to come to the health facility for screening; 3) contract referral, in which you and the counselor work together to notify your partner. Typically this means you will have 14 days to tell your partner after which the counselor will contact your partner and offer them STI testing/screening services; and 4) dual referral, in which the counselor or provider will sit with you and your partner and support you as you tell your partner about your STI. Which of these appeals the most to you? Why? What are your concerns about some of these notification strategies?
3. As part of this study, we are asking participants to give names and contact information of recent sexual partners. Is this something you think you would be willing or comfortable doing?
4. What do you think are some of the barriers to disclosing your partners’ names?
5. What are some of the potential benefits of disclosing their names? What might make you more or less likely to disclose partner names?
6. Some places use peers, so in your case, someone who was the same sex and roughly the same age, to help with the partner notification. These are people who have had training about confidentiality, but who are not themselves healthcare workers. How would you feel about having a partner notified by someone who was a “peer educator” rather than a healthcare worker?

*Probes*:
What might be some of the benefits of using a peer in this role?
Some of the drawbacks?

1. Would you tell your partner that you had an STI? What if you were no longer with that partner?
2. Have you ever ended a relationship because you thought you got an STI from a partner?

***Etiologic STI testing***

1. Have you ever had symptoms that you thought may be related to a sexually transmitted infection before today?

*Probes:*

Have you ever sought ever received care at this clinic before?

Where else do you go to get treatment (pharmacy, local healer, etc)?

What are some of the long-term complications of having a sexually transmitted infection?

1. What sort of symptoms do you typically associated with having a sexually transmitted infection?
2. Do you think you could have a sexually transmitted infection without having symptoms? Why or why not?

One thing proposed in this study will be testing for sexually transmitted infections using blood and urine. It is possible this will identify infections even when someone is not experiencing any symptoms.

1. How would you feel if you had a positive test result for a sexually transmitted infection without any symptoms?

*Probes:* Would you believe the result?

Do you think the possible long-term complications of sexually transmitted infections are different if there are no symptoms but a positive test?

What if the test result came back when you were having symptoms, would you be more likely to believe the result then?

1. We talked before about partner notification for sexually transmitted infections. Would having a diagnosis of an infection, without symptoms, change how likely you would be to notify your recent sexual partners?
2. We also talked earlier about your perceived risk of acquiring HIV infection. Would your perceived HIV risk be different if you had an asymptomatic sexually transmitted infection rather than a symptomatic sexually transmitted infection? Why or why not?
3. Do you think testing for sexually transmitted infections, even if someone is not having any symptoms, is important? For who?

*Probes:* Would having sexually transmitted infection testing, regardless of symptoms, provided as part of PrEP care be acceptable to you?
How would having a sexually transmitted infection diagnosis change your perceived need for PrEP?

***Long Acting Injectable PrEP***

Before we end the interview, I would like to talk to you briefly about a new kind of PrEP that has been studied around the world, including here in Malawi. In this kind of PrEP, instead of taking pills every day, you would receive injections once per month at the clinic. The injection would include similar drugs to what is in the oral pill, and they would remain in your body, helping to protect from HIV infection, for an entire month. You would likely need to come back to the clinic once per month to get the injections. These drugs have been studied in people without HIV for HIV prevention and were safe and effective. Once a person starts receiving the injections it is important that they come to the clinic every month, but, if they decide they don’t want to continue with the injections, they can go back to the daily pills. Injectable PrEP is not yet available for HIV prevention in most countries but may become available soon.

1. Just to begin, can you tell me some similarities and differences between daily oral ART and monthly injectable ART?

*Probes:* What do you know about their effectiveness? (Are they similar? Different?)
What do you know about how they are taken or administered?
What do you know about how often they are taken or administered?
What do you know about how often you must visit the clinic?

1. Have you ever heard of this type of PrEP before?

*Probes:* Where did you hear about it?
What sorts of things have you heard about it?

1. Based on what we’ve just discussed, what are your initial thoughts or feelings about monthly injectable PrEP?

*Probes*: Is there anything appealing to you about it?
What concerns do you have about it?

What questions would you want to ask about it?

1. Think about people you know, friends or other sexual partners. In your opinion, what would they think about monthly injectable PrEP?

*Probes*: What would the find more appealing about it compared with daily oral ART?

What might be some drawbacks about it compared with daily oral PrEP?

1. How does it make you feel that the medication is injected into the muscle?

*Probes*: Do you have any physical concerns? (HINT: pain, swelling, infection)

1. How do you feel about coming in on a monthly basis for an injection?

*Probes:* How often do you need to come to the clinic to get PrEP now?

What kinds of challenges would there be to changing your routine?

1. In what kind of setting would you want to these injections to be offered?

*Probes:* For example, would you want to come back to the STI clinic? Have the injection administered in the community? Some other clinical setting?
Would you feel comfortable if it was not a clinician giving you this injection?

1. Knowing what you now know, if you were given the choice of daily oral or monthly injectable PrEP, which would you prefer?

*Probes:* [IF YES/NO] What are a few main reasons you feel that way?

[IF UNSURE] What would help you decide?

[IF NO] Is there anything that would make you more amenable to monthly injectable PrEP?

1. (if deferred PrEP) If injectable PrEP were available at this clinic, do you think you would accept it?

***Closing interview***

Those are all of the questions that I have. Is there anything else you would like to share about your decision to use PrEP or your experience using it that we have not discussed?

Thank you for taking the time to talk with me. What questions do you have for me?
